# Cost-effectiveness of the combination of immunotherapy and chemotherapy for extensive-stage small-cell lung cancer: a systematic review

**DOI:** 10.1186/s12913-023-09727-7

**Published:** 2023-06-26

**Authors:** Tao Wang, Yilin Li, Xiaoqiang Zheng

**Affiliations:** 1 School of Economics and Management, Southwest Petroleum University, Chengdu, Sichuan Province China; 2 Department of Gastroenterology, Hospital of Chengdu University of Traditional Chinese Medicine, Chengdu, Sichuan Province China

**Keywords:** Extensive-stage small-cell lung cancer, Cost-effectiveness, Healthcare cost, Immunotherapy combined with chemotherapy, Immune checkpoint inhibitors

## Abstract

**Background:**

The combination of immunotherapy and chemotherapy for extensive-stage small-cell lung cancer (ES-SCLC) was primarily carried out with a combination of immune checkpoint inhibitors (ICIs) and platinum-etoposide (EP). It is likely to be more effective in treating ES-SCLC than EP alone, but could result in high healthcare costs. The study aimed to investigate the cost-effectiveness of this combination therapy for ES-SCLC.

**Methods:**

We searched literature from the following databases: PubMed, Embase, Cochrane Library, and Web of Science for studies on cost-effectiveness of immunotherapy combined with chemotherapy for ES-SCLC. The literature search period was up to April 20, 2023. The quality of the studies was evaluated using the Cochrane Collaboration's tool and Consolidated Health Economic Evaluation Reporting Standards (CHEERS) checklist.

**Results:**

A total of 16 eligible studies were included in the review. All studies met CHEERS recommendations, and all randomized controlled trials (RCTs) in these studies were rated as having low risk of bias using the Cochrane Collaboration's tool. The treatment regimens compared were ICIs plus EP or EP alone. All studies mainly used incremental quality-adjusted life year and incremental cost-effectiveness ratio as outcomes. Most ICIs plus EP treatment regimens were not cost-effective based on corresponding willingness-to-pay thresholds.

**Conclusions:**

Adebrelimab plus EP and serplulimab plus EP were probably cost-effective for ES-SCLC in China, and serplulimab plus EP was probably cost-effective for ES-SCLC in the U.S. Lowering the price of ICIs and selecting ES-SCLC patients who were sensitive to ICIs could improve the cost-effectiveness of the ICIs-combined treatment.

**Supplementary Information:**

The online version contains supplementary material available at 10.1186/s12913-023-09727-7.

## Introduction

Lung cancer is the most prevalent cancer and the leading cause of cancer-related mortality globally [1]. Small-cell lung cancer (SCLC) accounts for around 10–15% of lung cancer cases with a high rate of early metastasis (approximately 60–70%) [2, 3]. Furthermore, about two-thirds of SCLC patients suffer from extensive-stage small-cell lung cancer (ES-SCLC) [4]. The overall survival (OS)of the patients is dependent on the early detection, with a 5-year survival rate of 20–25% for localized-stage SCLC (LS-SCLC) and 2% for ES-SCLC [5, 6]. Therefore, it is important to focus on the treatment of ES-SCLC.

There have been few alternatives to platinum-etoposide (EP) chemotherapy as the first-line therapy for ES-SCLC over the past several decades [3, 7, 8]. Even though ES-SCLC is sensitive to chemotherapy with EP, nearly all patients develop drug resistance and undergo tumor relapse within six months with an objective response rate of 50–60% [9]. With no major discoveries in medical interventions and no progress in patient outcomes over the past twenty years, the invention of immune checkpoint inhibitors (ICIs), such as inhibitors of programmed cell death protein 1 (PD-1) and programmed death-ligand 1 (PD-L1), is a welcome relief to promote immunotherapy and improve survival in ES-SCLC patients. The discovery of ICIs has replaced EP treatment regimen as the primary therapy for ES-SCLC patients [10, 11]. Some studies have demonstrated that combining ICIs, such as durvalumab (PD-L1), atezolizumab (PD-L1), and pembrolizumab (PD-1), with EP could greatly increase OS in patients than EP alone. Therefore, combining ICIs with EP has become an alternative option for the treatment of ES-SCLC.

The combination of immunotherapy and chemotherapy indicates significant progress in medical intervention to treat ES-SCLC with increasing demands for this treatment regimen [12]. In addition to the clinical benefits and toxicity, the cost has become an increasingly important factor for cancer treatment [13]. Therefore, greater emphasis should be placed on the economic implications of immunotherapy combined with chemotherapy.

## Methods

This study was a systematic review of cost-effectiveness analyses. The risk of bias assessment was performed using the evaluation criteria specific to not only the cost-effectiveness analyses but also the randomized controlled trials (RCTs), since both the risk of bias in cost-effectiveness analyses and the risk of bias in RCTs of the included studies could affect the results.

This study was carried out based on the Preferred Reporting Items for Systematic Reviews and Meta-Analyses (PRISMA)Statement [14], and the Criteria for Cost(-Effectiveness) Review Outcomes (CiCERO) by the International Society for Pharmacoeconomics and Outcomes Research (ISPOR) [15]. Our systematic review protocol was registered with PROSPERO (registration number: CRD42022313621).

### Search strategy

A researcher systematically searched PubMed, Embase, Web of Science, and the Cochrane Library for publications up to April 20, 2023, using predefined keywords and Medical Subject Headings (MeSH) terms, including “Chemotherapy”, “ES-SCLC”, “cost-effectiveness”, and their synonyms to obtain relevant literature on the cost-effectiveness of immunotherapy combined with chemotherapy for patients with ES-SCLC. Furthermore, potential candidate papers were manually checked in the references of the included studies. This study requires no ethical approval or patient consent. The detailed search strategy is shown in the supplementary materials.

### Inclusion and exclusion criteria

The inclusion criteria were as follows: (1) Eligible patients were at least 18 years old with treatment-naïve, histologically or cytologically documented ES-SCLC; (2) the treatment regimen was immunotherapy combined with chemotherapy; (3) Economic evaluations (cost-effectiveness analyses, cost-utility analyses, and cost–benefit analyses) were provided in the studies if both the costs and expected benefits were presented for each analytical approach; (4) the full-texts of the included studies were written in English; (5) the types of trials in the included literature were RCTs.

The exclusion criteria were as follows: (1) studies that provide no economic analysis; (2) duplicated studies; (3) reviews, case reports, conference abstracts, letters to the editor, and other nonclinical literature; (4) published studies written in non-English language; (5) studies on non-human research.

### Review of study selection

Two researchers (TW and YLL) independently examined the eligibility of the studies, and selected the titles and abstracts of all identified potential studies, followed by a full-text review to finalize the study selection. The discrepancy between the two researchers, if any, was resolved with a third reviewer (XQZ).

### Data extraction, determining of cost-effectiveness and quality assessment

Data were extracted from each eligible study by two researchers (TW and YLL). Any discrepancies were resolved through discussion with a third reviewer (XQZ) to ensure the validity of the research results. For each study, the relevant information was recorded in 2 tables, including author, year of publication, perspective, estimated total costs, life-years (LYs), quality-adjusted lifeyear (QALY), country, ICIs, incremental QALY, incremental cost-effectiveness ratio (ICER), willingness-to-pay (WTP) threshold, and the proposed price reduction for ICIs.

The cost-effectiveness of ICIs plus EP was mainly demonstrated through comparing the ICER with the WTP threshold. QALY was an adjusted life expectancy used to evaluate and compare the combined effects of health interventions, and it reflected the combined real value of a therapeutic intervention. In this review, data on QALY was required to obtain the incremental QALY, which represented whether there was a positive therapeutic effect of ICIs plus EP compared with EP alone or a positive therapeutic effect compared between different ICIs plus EP. Furthermore, there is no specific standard for WTP threshold. The WTP threshold in the U.S. is generally $100,000/QALY or $150,000/QALY [16], and WTP threshold in China is generally calculated as three times the country's GDP per capita for the year, as suggested by the World Health Organization [17].

Two researchers (TW and YLL) independently evaluated the quality of the included studies and their randomized trials using the Consolidated Health Economic Evaluation Reporting Standards (CHEERS) [18] checklist and the Cochrane Collaboration's tool [19], and any inconsistencies or disputes were settled with a third independent reviewer (XQZ). The CHEERS checklist developed for the reporting of health economic assessment, contains 28 items divided into seven categories: (1) title, (2) abstract, (3) introduction, (4) methods, (5) results, (6) discussion, and (7) other relevant information. The Cochrane Collaboration's tool was used to evaluate the risk of bias of the randomized trials in six distinct domains and seven sub-items. Judgments of all domains can result in "low risk of bias", "unclear risk of bias", and "high risk of bias".

## Results

### Study selection

**Fig. 1:**
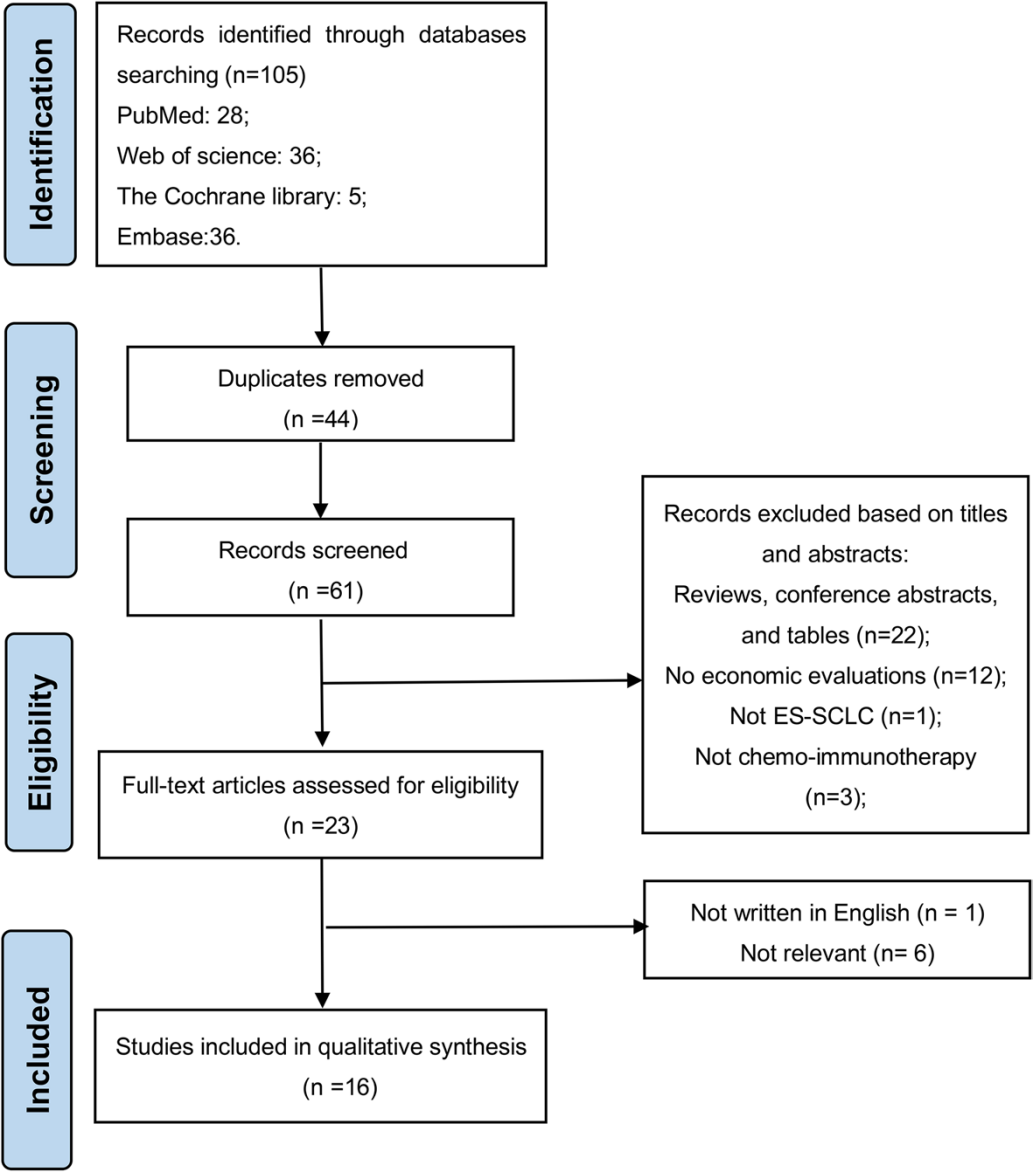
Flow diagram of study inclusion

Records from the results of all retrieved search were downloaded and merged with Endnote version X9. After the duplicates were excluded, all titles and abstracts were reviewed for potentially eligible studies, and the full texts of the potentially eligible studies were read for verification of their eligibility. A total of 105 potentially relevant studies were initially identified through database search (PubMed n = 28, Embase n = 36, the Cochrane Library n = 5, Web of Science n = 36). After 44 duplicates were excluded through the initial assessment, the titles, abstracts, and full texts of the remaining studies were screened, and 45 more articles were excluded. Ultimately, 16 studies published between January 2019 and April 2023 were included. A flow chart of the literature identification process is illustrated in Fig. 1.

### Characteristics of the studies

Summary of the basic characteristics of the 16 included studies [20–35] is presented in Table 1. The clinical sample data of these studies were from seven published RCTs (IMpower 133 [36], CASPIAN [37], KEYNOTE-604 [38], CA184-156 [39], EA5161 [40], ASTRUM-005 [41], and CAPSTONE-1 [42]). All studies from China reported the cost in US$, which was converted from RMB using the RMB to US$ exchange rate. The analyses were performed from a third-party payer perspective (10/16, 62.5%) in most studies, and from a societal perspective (2/16, 12.5%) or a health-care system perspective (4/16, 25.0%) in the rest of the studies. For all the included studies, the interventions were ICIs plus EP, with the ICIs being atezolizumab, durvalumab, nivolumab, ipilimumab, pembrolizumab, adebrelimab, and serplulimab, respectively, and were compared with EP alone. All the 16 studies used Markov models (12/16, 75.0%) and partitioned survival models (4/16, 25.0%) to assess the cost-effectiveness of various interventions. Of these studies, three [20, 21, 27]evaluated the cost-effectiveness of atezolizumab plus EP (AEP), five [22–24, 29, 30] assessed the cost-effectiveness of durvalumab plus EP (DEP), two assessed [26, 28] the cost-effectiveness of pembrolizumab plus EP (PEP), one [33] assessed the cost-effectiveness of adebrelimab plus EP (ADEP), two [34, 35] assessed the cost-effectiveness of serplulimab plus EP (SEP), two [25, 32] assessed the cost-effectiveness of AEP and DEP, and one [31] assessed the cost-effectiveness of AEP, DEP, PEP, nivolumab plus EP (NEP), and ipilimumab plus EP (IEP). The maximum estimated total cost of AEP was $160,219.00, and the minimum was $48,129.00; the maximum estimated total cost of DEP was $187,503.00, and the minimum was $41,106.00; the maximum estimated total cost of PEP was $130,692.00, and the minimum was $72,012.27; the maximum estimated total cost of SEP was $107,558.01, and the minimum was $11,202.00; the estimated total cost of NEP, IEP and ADEP was $87,897.01, $249,215,23 and $25,312.00, respectively. For AEP, the maximum life expectancy of the patient was 1.54 years, and the minimum was 1.11 years; for DEP, the maximum life expectancy of patients was 2.20 years, and the minimum was 0.99 years; for PEP, the maximum life expectancy of patients was 1.83 years, and the minimum was 1.43 years; for SEP, the maximum life expectancy of patients was 2.243 years, and the minimum was 2 years; for NEP, the life expectancy of patients was 1.60 years; for IEP, the life expectancy of patients was 1.18 years; for ADEP, the life expectancy of patients was 2.47 years.

**Table 1:** Study characteristics

Authors (year of publication)	Perspective	Estimated total costs (US$)	Life years	Analysis model
Li et al. (2019) [20]	The Chinese perspective	AEP: 48,129.00; EP: 12,920.00	/	Markov model
Zhou et al. (2019) [21]	The American perspective	AEP: 83,439.00; EP: 30,558.00	/	Markov model
Zhang et al. (2020) [22]	The US payers	DEP: 90,072.83; EP: 11,874.08	DEP: 0.99; EP: 0.57	Partitioned survival model
Ding et al. (2021) [23]	The US health- care system	DEP: 164,508.07; EP: 73,038.11	DEP: 2.20; EP: 1.93	Markov model
Lin et al. (2021) [24]	The US payers	DEP: 134,322.00; EP: 38,414.00	DEP: 1.73; EP: 0.87	Markov model
Liu et al. (2021) [25]	The US payers	DEP: 92,391.00; AEP: 86,655.00; EP: 24,582.00	/	Markov model
Liu et al. (2021) [26]	The US payers	PEP: 126,362.00; EP: 44,890.00	PEP: 1.43; EP: 1.13	Markov model
Wang et al. (2021) [27]	The US payers	AEP: 109,051.00 (mixture cure model); AEP: 109,824.00 (standard parametric model); EP: 25,556.00	AEP: 1.12 (mixture cure model); AEP: 1.11 (standard parametric model); EP: 0.96	Partitioned survival model
Zhu et al. (2021) [28]	The US payers	PEP: 130,692.00; EP: 17,067.00	PEP: 1.83; EP: 1.51	Markov model
Liu et al. (2022) [29]	The Chinese health-care system	DEP: 90,555.00 (Without Patient Assistance Program); DEP: 62,885.00 (With Patient Assistance Program); EP: 14,201.00	DEP: 1.86; EP: 1.34	Markov model
Tong et al. (2022) [30]	The Chinese payers	DEP: 41,106.00; EP: 8,886.00	/	Markov model
Kang et al. (2021) [31]	The Chinese health-care system	PEP: 72,012.27 DEP: 90,750.92 AEP: 41,194.22 NEP: 87,897.01 IEP: 249,215,23	PEP:1.34 DEP:1.45 AEP:1.54 NEP:1.60 IEP:1.18	Partitioned survival model
Ionova et al. (2022) [32]	The US payers	AEP: 160,219.00 DEP: 187,503.00	/	Markov model
You et al. (2022) [33]	The Chinese health-care system	ADEP: 25,312.00 EP: 14,846.00	ADEP: 2.47 EP: 1.59	Markov model
Zhu et al. (2022) [34]	The Chinese payers	SEP: 11,202.00 EP: 7,194.00	SEP: 2.243 EP: 1.661	Markov model
Shao et al. (2023) [35]	The Chinese payers	SEP: 33,616.66 EP: 14,247.49	SEP: 2 EP: 1.13	Partitioned survival model
	The US payers	SEP: 107,558.01 EP: 42,639.65		

*SCLC* small-cell lung cancer, *ES-SCLC* extensive-stage small-cell lung cancer, *EP* platinum-etoposide, *AEP* atezolizumab plus platinum-etoposide, *DEP* durvalumab plus platinum-etoposide, *PEP* pembrolizumab plus platinum-etoposide, *NEP* nivolumab plus platinum-etoposide, *IEP* ipilimumab plus platinum-etoposide, *ADEP* adebrelimab plus platinum-etoposide, *SEP* serplulimab plus platinum-etoposide, *QALY* quality-adjusted life-years

### Results of the quality assessment

**Fig. 2:**
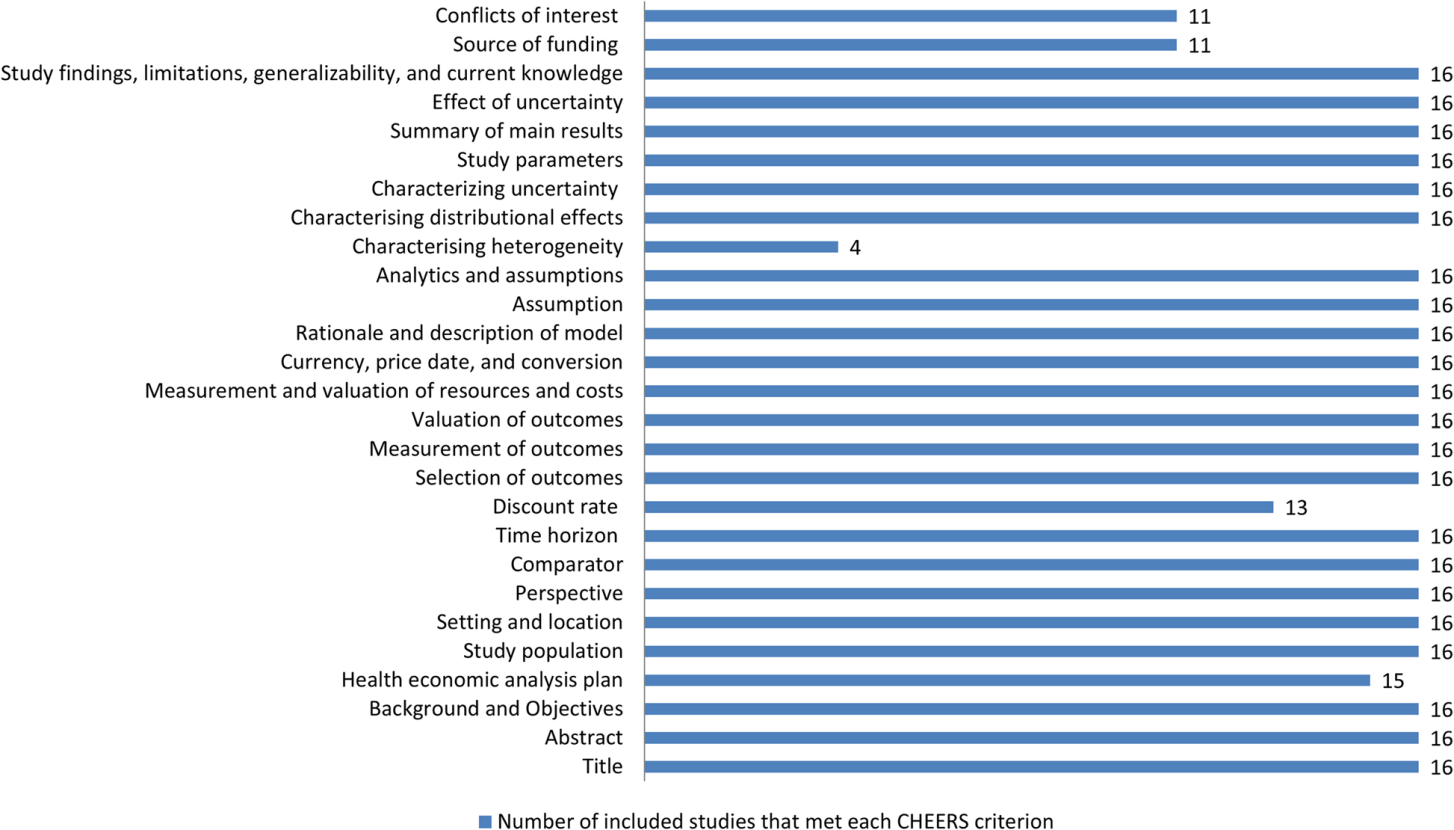
Number of included studies that met each CHEERS criterion

Based on the CHEERS checklist, all the included studies presented good reporting quality. The percentages of items met ranged between 82.14% and 92.86%. The least frequently reported item in the included studies was “characterizing heterogeneity,” followed by “conflicts of interest” and “source of funding.” More details are shown in Table 2 and Fig. 2.

**Table 2:** The CHEERS 2022 checklist for study appraisal

Checklist item			Included studies															
**Section/topic**	**no**	**Guidance for reporting**	**Li et al. (2019)** [20]	**Zhou et al. (2019)** [21]	**Zhang et al. (2020)** [22]	**Ding et al. (2021)** [23]	**Lin et al. (2021)** [24]	**Liu et al. (2021)** [25]	**Liu et al. (2021)** [26]	**Wang et al. (2021)** [27]	**Zhu et al. (2021)** [28]	**Liu et al. (2022)** [29]	**Tong et al. (2022)** [30]	**Kang et al. (2021)** [31]	**Ionova et al. (2022)** [32]	**You et al. (2022)** [33]	**Zhu et al. (2022)** [34]	**Shao et al. (2023)** [35]
Title																		
Title	1	Identify the study as an economic evaluation and specify the interventions being compared	√	√	√	√	√	√	√	√	√	√	√	√	√	√	√	√
Abstract																		
Abstract	2	Provide a structured summary that highlights context, key methods, results, and alternative analyses	√	√	√	√	√	√	√	√	√	√	√	√	√	√	√	√
Introduction																		
Background and objectives	3	Give the context for the study, the study question, and its practical relevance for decision making in policy or practice	√	√	√	√	√	√	√	√	√	√	√	√	√	√	√	√
Methods																		
Health economic analysis plan	4	Indicate whether a health economic analysis plan was developed and where available	√	√	√	√	√	√	√	√	√	√	√	√	√	×	√	√
Study population	5	Describe characteristics of the study population (such as age range, demographics, socioeconomic, or clinical characteristics)	√	√	√	√	√	√	√	√	√	√	√	√	√	√	√	√
Setting and location	6	Provide relevant contextual information that may influence findings	√	√	√	√	√	√	√	√	√	√	√	√	√	√	√	√
Comparators	7	Describe the interventions or strategies being compared and why chosen	√	√	√	√	√	√	√	√	√	√	√	√	√	√	√	√
Perspective	8	State the perspective(s) adopted by the study and why chosen	√	√	√	√	√	√	√	√	√	√	√	√	√	√	√	√
Time horizon	9	State the time horizon for the study and why appropriate	√	√	√	√	√	√	√	√	√	√	√	√	√	√	√	√
Discount rate	10	Report the discount rate(s) and reason chosen	×	×	×	√	√	√	√	√	√	√	√	√	√	√	√	√
Selection of outcomes	11	Describe what outcomes were used as the measure(s) of benefit(s) and harm(s)	√	√	√	√	√	√	√	√	√	√	√	√	√	√	√	√
Measurement of outcomes	12	Describe how outcomes used to capture benefit(s) and harm(s) were measured	√	√	√	√	√	√	√	√	√	√	√	√	√	√	√	√
Valuation of outcomes	13	Describe the population and methods used to measure and value outcomes	√	√	√	√	√	√	√	√	√	√	√	√	√	√	√	√
Measurement and valuation of resources and costs	14	Describe how costs were valued	√	√	√	√	√	√	√	√	√	√	√	√	√	√	√	√
Currency, price date, and conversion	15	Report the dates of the estimated resource quantities and unit costs, plus the currency and year of conversion	√	√	√	√	√	√	√	√	√	√	√	√	√	√	√	√
Rationale and description of model	16	If modelling is used, describe in detail and why used. Report if the model is publicly available and where it can be accessed	√	√	√	√	√	√	√	√	√	√	√	√	√	√	√	√
Analytics and assumptions	17	Describe any methods for analysing or statistically transforming data, any extrapolation methods, and approaches for validating any model used	√	√	√	√	√	√	√	√	√	√	√	√	√	√	√	√
Characterising heterogeneity	18	Describe any methods used for estimating how the results of the study vary for subgroups	×	×	×	√	×	×	×	×	√	×	×	×	×	√	×	√
Characterising distributional effects	19	Describe how impacts are distributed across different individuals or adjustments made to reflect priority populations	√	√	√	√	√	√	√	√	√	√	√	√	√	√	√	√
Characterising uncertainty	20	Describe methods to characterise any sources of uncertainty in the analysis	√	√	√	√	√	√	√	√	√	√	√	√	√	√	√	√
Approach to engagement with patients and others affected by the study	21	Describe any approaches to engage patients or service recipients, the general public, communities, or stakeholders (such as clinicians or payers) in the design of the study	Not applicable	Not applicable	Not applicable	Not applicable	Not applicable	Not applicable	Not applicable	Not applicable	Not applicable	Not applicable	Not applicable	Not applicable	Not applicable	Not applicable	Not applicable	Not applicable
Results																		
Study parameters	22	Report all analytic inputs (such as values, ranges, references) including uncertainty or distributional assumptions	√	√	√	√	√	√	√	√	√	√	√	√	√	√	√	√
Summary of main results	23	Report the mean values for the main categories of costs and outcomes of interest and summarise them in the most appropriate overall measure	√	√	√	√	√	√	√	√	√	√	√	√	√	√	√	√
Effect of uncertainty	24	Describe how uncertainty about analytic judgements, inputs, or projections affect findings. Report the effect of choice of discount rate and time horizon, if applicable	√	√	√	√	√	√	√	√	√	√	√	√	√	√	√	√
Effect of engagement with patients and others affected by the study	25	Report on any difference patient/service recipient, general public, community, or stakeholder involvement made to the approach or findings of the study	Not applicable	Not applicable	Not applicable	Not applicable	Not applicable	Not applicable	Not applicable	Not applicable	Not applicable	Not applicable	Not applicable	Not applicable	Not applicable	Not applicable	Not applicable	Not applicable
Discussion																		
Study findings, limitations, generalisability, and current knowledge	26	Report key findings, limitations, ethical or equity considerations not captured and how these could affect patients, policy, or practice	√	√	√	√	√	√	√	√	√	√	√	√	√	√	√	√
Other relevant information																		
Source of funding	27	Describe how the study was funded and any role of the funder in the identification, design, conduct, and reporting of the analysis	√	√	√	√	×	√	×	×	√	×	√	×	√	√	√	√
Conflicts of interest	28	Report authors’ conflicts of interest according to journal or International Committee of Medical Journal Editors requirements	√	√	×	√	×	×	×	×	√	√	√	√	√	√	√	√
Total number of items met			24	24	23	26	23	24	23	23	26	24	25	24	25	25	26	26
Percentage of items met			85.71%	85.71%	82.14%	92.86%	82.14%	85.71%	82.14%	82.14%	92.86%	85.71%	89.29%	85.71%	89.29%	89.29%	92.86%	92.86%

**Fig. 3:**
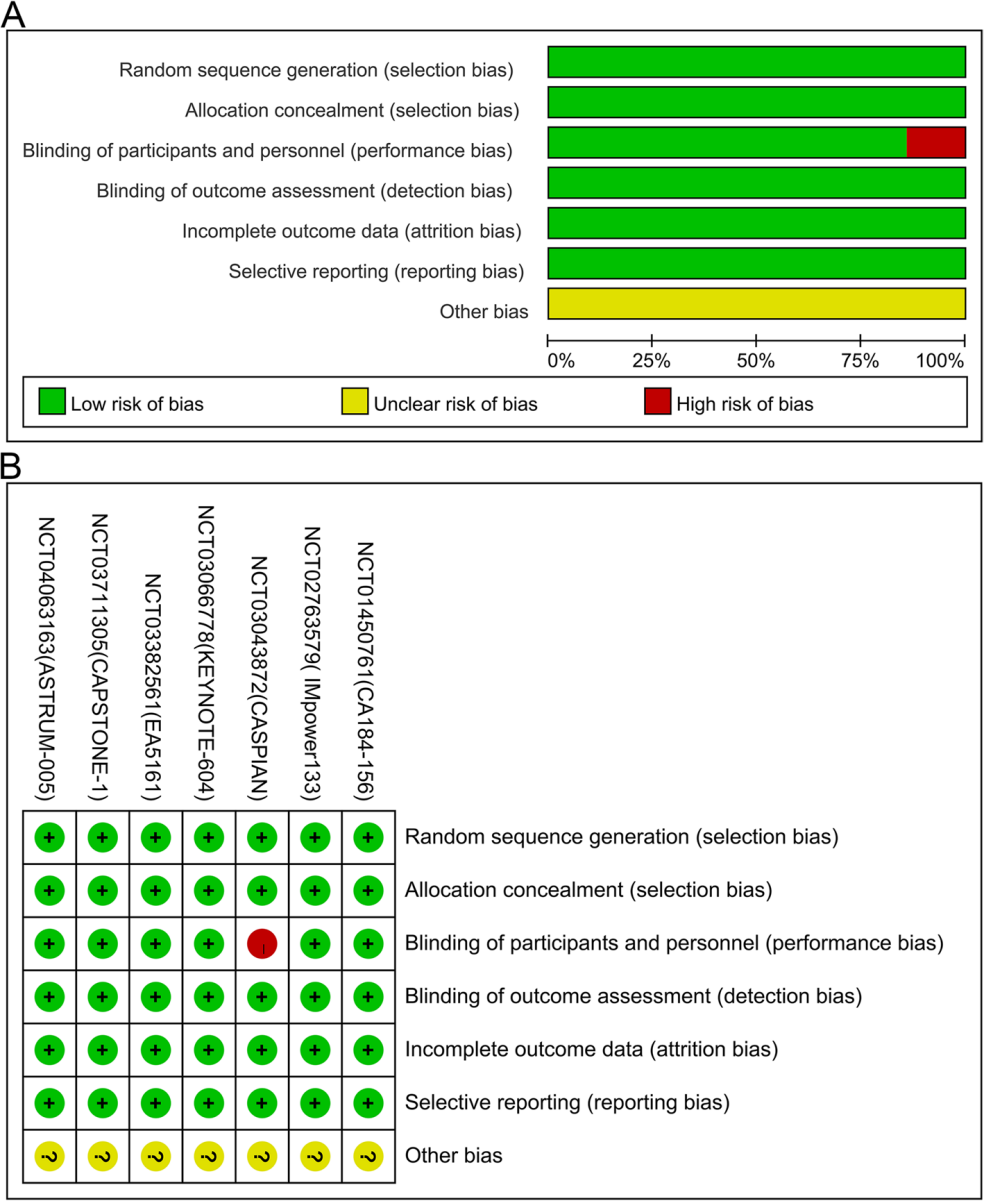
**a** Risk of Bias Graph; **b** Risk of Bias Summary Table

All included studies used clinical sample data from seven randomized trials [36–42], namely IMpower 133, CASPIAN, KEYNOTE-604, CA184-156, EA5161, ASTRUM-005, and CAPSTONE-1. It is believed that in addition to the quality assessment of the included studies, a quality assessment of the trials was also required. Therefore, a quality assessment of the seven trials was conducted in this review to assess the risk of bias. The results suggested that all seven trials had "unclear risk of bias" in terms of "other bias." This was because they all had sponsors. Based on the available information, we could not determine whether the sponsors had influenced the researchers to make findings in favor of the sponsors. Furthermore, the CASPIAN [37] had a "high risk of bias" in terms of "blinding of outcome assessment (performance bias)" because it was an open-label trial, which could affect study conduct and the outcome assessment. All RCTs in the included studies had an overall low risk of bias in terms of quality assessment. Further information about the risk of bias assessment is described in Fig. 3.

### Types of modeling approaches and health states

Cancer study frequently employs Markov and partitioned survival (PS) models to calculate long-term costs and effects [43]. The structure of the PS model resembles the Markov model. In contrast to a Markov model, which requires transition probabilities between any two health states, the PS model uses proportions of patients in each health state at every time point [44]. Markov and PS models were employed in all the included studies. The primary endpoint measures of the models included the total costs, LYs, QALY, and ICER. These model-based studies similarly used three key health states: progression-free survival (PFS), progressed disease (PD), and death [45]. All patients were included in the model in the PFS health states and could undergo a transition to progressive disease and death. The parametric model was employed in all the included studies that constructed the Markov and PS models for PFS and OS. All models extended the time horizon beyond the observed data and incorporated an exploration of the impact of choosing a particular parametric model, and five survival distributions (Weibull, Log-logistic, Log-normal, Gamma, and Exponential) were used to parameterize the models. The best-fitting parametric distribution was chosen using statistical tests according to the combination of visual inspection and the Akaike and Bayesian indicator [46]. Importantly, it is uncommon to consider the choice of model structure in published studies. However, it can affect the analysis results [47]. Many of the included studies demonstrated good modeling practices, but few studies described how to select a specific model that fitted into the study.

### Cost-effectiveness outcomes

The incremental QALY of ICIs vs. EP was greater than 0, suggesting that they had clinical benefit, and the ICERs of ICIs in most included studies [20–30, 32] were much greater than their respective corresponding WTP threshold. This seemed to indicate that the combination of immunotherapy and chemotherapy was not a better cost-effective option. However, three studies [33–35] indicated that adebreumab and sepolizumab were cost-effective in China when the WTP was $37,653.00 and $38,184.00, respectively. SEP could also be cost-effective in the U.S. when the WTP was $150,000.00. Furthermore, one [31] of the included studies suggested that AEP was probably more cost-effective than DEP, NEP, IEP, and PEP in China when the WTP threshold was $31,313, but it did not report whether AEP was more cost-effective compared with EP alone. More details about the cost-effectiveness outcomes are presented in Table 3.

**Table 3:** Cost-effectiveness outcomes

Authors (year)	Country	ICIs of the included studies	QALY	Incremental QALY	ICER (US$/QALY)	WTP threshold(US$/QALY)	The proposed price reduction for ICIs
Li et al. (2019) [20]	China	Atezolizumab	AEP: 0.858 EP: 0.786	AEP vs. EP: 0.072	AEP vs. EP: 489,013.00	25,929.00	The price would be reduced by more than 80%
Zhou et al. (2019) [21]	America	Atezolizumab	DEP: 0.60 EP: 0.50	AEP vs. EP: 0.10	AEP vs. EP: 528,810.00	100,000.00	NR
Zhang et al. (2020) [22]	America	Durvalumab	DEP: 0.55 EP: 0.33	DEP vs. EP: 0.22	DEP vs. EP: 355,448.86	100,000.00 or 150,000.00	WTP = $100,000.00/QALY, the price would be reduced by 70%; WTP = $150,000.00/QALY, the price would be reduced by 50%
Ding et al. (2021) [23]	America	Durvalumab	DEP: 1.45 EP: 1.25	DEP vs. EP: 0.20	DEP vs. EP: 464,711.90	150,000.00	NR
Lin et al. (2021) [24]	America	Durvalumab	DEP: 0.93 EP: 0.49	DEP vs. EP: 0.44	DEP vs. EP: 216,953.00	150,000.00	The price would be reduced by 30.70%
Liu et al. (2021) [25]	America	Atezolizumab and duralumab	AEP: 0.74 DEP: 0.724 EP: 0.578	AEP vs. EP: 0.162; DEP vs. EP: 0.146 AEP vs. DEP: 0.016	AEP vs. EP: 382,469.00; DEP vs. EP: 464,593.00 DEP vs. AEP: Dominated^a^	100,000.00	Atezolizumab: the price would be reduced by more than 77%; duralumab: the price would be reduced bymore than 80%
Liu et al. (2021) [26]	America	Pembrolizumab	PEP: 0.55 EP: 0.44	PEP vs. EP: 0.11	PEP vs. EP: 334,373.00	100,000.00	The price would be reduced by65%
Wang et al. (2021) [27]	America	Atezolizumab	Mixture cure model for AEP: 0.74; standard parametric model for AEP: 0.73 EP: 0.63	Mixture cure model for AEP vs. EP: 0.11; standard parametric model for AEP vs. EP: 0.10	Mixture cure model for AEP vs. EP:785,848.00; standard parametric model for AEP vs. EP:827,610.00	100,000.00	NR
Zhu et al. (2021) [28]	America	Pembrolizumab	PEP: 1.07 EP: 0.89	PEP vs. EP: 0.18	PEP vs. EP: 647,509.00	150,000.00	The price would be reduced by 80.30%
Liu et al. (2022) [29]	China	Durvalumab	DEP: 0.96 EP: 0.71	Without Patient Assistance Program: DEP vs. EP: 0.25; With Patient Assistance Program: DEP vs. EP: 0.25	Without Patient Assistance Program: DEP vs. EP: 302,051.00; With Patient Assistance Program: DEP vs. EP: 192,591.00	30,828.00	The price would be reduced by 90%
Tong et al. (2022) [30]	China	Durvalumab	DEP: 0.63 EP: 0.49	DEP vs. EP: 0.14	DEP vs. EP: 230,142.90	28,527.00	NR
Kang et al. (2021) [31]	China	Atezolizumab, duralumab, pembrolizumab,nivolumab, and ipilimumab	PEP: 0.75 DEP: 0.79 AEP: 0.83 NEP:0.88 IEP: 0.66	/	DEP vs.PEP: 469,482.10; NEP vs.PEP: 119,234.60 IEP vs.PEP: Dominated^a^ PEP vs.AEP: Dominated^a^	31,313.00	Nivolumab: the price would be reduced by 80%; atezolizumab: it would be cost-effective
Ionova et al. (2022) [32]	America	Atezolizumab and duralumab	AEP: 1.08 DEP: 0.91	AEP vs. DEP: 0.17	DEP vs.AEP: 165,182.00	150,000.00	Duralumab: the price would be reduced by13%
You et al. (2022) [33]	China	Adebrelimab	ADEP: 1.21 EP: 0.81	ADEP vs. EP: 0.40	ADEP vs.EP: 25,914.00	37,653.00	Cost-effective
Zhu et al. (2022) [34]	China	Serplulimab	SEP: 1.217 EP: 0.885	SEP vs. EP: 0.332	SEP vs. EP: 12,077	37,653.00	Cost-effective
Shao et al. (2023) [35]	China	Serplulimab	SEP: 1.39 EP: 0.81	SEP vs. EP: 0.58	SEP vs. EP: 33,392.41	38,184.00	Cost-effective
	America		SEP: 1.42 EP: 0.82	SEP vs. EP: 0.60	SEP vs. EP: 106,756.95	100,000.00 or 150,000.00	It would be cost-effective when the WTP was $150,000.00/QALY in America

*WTP* willingness-to-pay, *QALY* quality-adjusted life-years, *ICER* Incremental cost-effectiveness ratio, *EP* platinum-etoposide, *AEP* atezolizumab plus platinum-etoposide, *DEP* durvalumab plus platinum-etoposide, *PEP* pembrolizumab plus platinum-etoposide, *NEP* nivolumab plus platinum-etoposide, *IEP* ipilimumab plus platinum-etoposide, *ADEP* adebrelimabplus platinum-etoposide, *SEP* serplulimab plus platinum-etoposide, *ICI* Immune checkpoint inhibitor, Dominated

^a^showed lower effectiveness and higher cost, *NR* not reported

Moreover, Wang et al. [27] established a mixture cure model and a standard parametric model to analyze AEP, and indicated that AEP would provide patients in the intervention group with significant long-term survival benefits when using the mixture cure model rather than the standard parametric model. The total cost in mixture cure model was lower, the total QALY and Life Year Gained (LYG) were higher, and the ICER in the mixture cure model was lower than that in the standard parametric model. Therefore, a comparison of the mixture cure model compared with a standard parametric survival model resulted in estimates that AEP were more cost-effective. Liu et al. [29] carried out a scenario analysis of the patient assistance program (donation of high-cost drugs to specific patients to improve their quality of life and reduce their financial burden) for durvalumab, and they found that the cost-effectiveness ratio would be higher than without the patient assistance program.

### Sensitivity analysis

Sensitivity analysis such as probabilistic sensitivity analysis and one-way deterministic sensitivity analysis was employed to evaluate the uncertainty of the model, and the tornado diagram, as the most common display diagram for sensitivity analysis, was plotted. This analysis investigated the robustness of a model's outcomes when inputs change and assessed the model's sensitivity to changes in each key model parameter [48]. All of the included studies employed one-way sensitivity analysis and probabilistic sensitivity analysis.

The one-way sensitivity analysis indicated that the price of ICIs was an essential and prevalent influencing factor in these studies. Furthermore, the probabilistic sensitivity analysis of most included studies [20–30, 32] indicates that ICIs plus chemotherapy were not cost-effective, with the probability of being cost-effective between 0 and 53% under existing WTP thresholds. However, the probabilistic sensitivity analysis of one [31] of the included studies showed that atezolizumab had a 99.7% probability of cost-effectiveness compared with durvalumab, nivolumab, ipilimumab, and pembrolizumab in China when the WTP was $31,313. Also, three studies [33–35] indicated adebrelimab had an 89.1% probability of cost-effectiveness, and serplulimab had a probability of cost-effectiveness not less than 91.6% in China when the WTP was $37,653.00 and $38,184.00, respectively.

Nine [20, 22, 24–26, 28, 29, 31, 32] of the included studies proposed price reductions of ICIs that were to make the ICIs cost-effective. Under American or Chinese WTP thresholds, the maximum proposed price reduction for durvalumab was 90%, 80.30% for pembrolizumab, 80% for nivolumab, and 80% for atezolizumab [17].

To sum up, the price of ICIs was an important factor that affected the cost-effectiveness of immunotherapy combined with chemotherapy for ES-SCLC.

## Discussion

This review evaluated and summarized the current state of the level of evidence regarding the cost-effectiveness of immunotherapy combined with chemotherapy for ES-SCLC. To the best of our knowledge, this study was the first systematic review discussing the cost-effectiveness of immunotherapy combined with chemotherapy for ES-SCLC. Due to the relatively small amount of new clinical evidence for the use of immunotherapy combined with chemotherapy for ES-SCLC, the economic evaluations has rarely been discussed. The results of most included studies [20–30, 32] suggested that the combination of immunotherapy with chemotherapy was not cost-effective compared with chemotherapy alone. However, three [33–35] of the included studies suggested that ADEP and SEP were probably cost-effective in China, and SEP could also be cost-effective in the U.S. when the WTP was $150,000.00. It is probably because the price of adebrelimab confers a great advantage over other PD-L1 inhibitors imported from abroad, as it is an indigenously developed PD-L1 inhibitor in China. For ADEP and SEP, it may be attributable to the patient assistance program making atezolizumab and serplulimab affordable in China, which can reduce patients' financial burden [31, 33]. Meanwhile, China's per capita GDP is increasing with the development of the economy, making the WTP increases accordingly. The above two factors may increase the probability of ADEP and SEP being cost-effective in China. Furthermore, one [31] of the included studies suggested that AEP was probably more cost-effective than DEP, NEP, IEP, and PEP in China when the WTP threshold was $31,313, but it did not report whether AEP was more cost-effective compared with EP alone.

The innovative combination therapy of ICIs and chemotherapy has significantly changed the treatment strategy for ES-SCLC, causing great concerns among oncologists and patients. Seven clinical trials evaluated the efficacy of AEP, DEP, NEP, IEP, PEP, ADEP, and SEP for ES-SCLC, and showed favorable clinical outcomes of the seven strategies. Based on these trials, the 16 studies used Markov and PS models for cost-effectiveness analyses of AEP, DEP, NEP, IEP, PEP, ADEP, and SEP for ES-SCLC from the standpoints of payers, society, and the health-care system in the U.S. or China. As computing power and appreciation of modeling approaches have increased, many scholars use Markov and PS models in their studies. It demonstrates greater awareness of modeling techniques and superior treatments that extend patient survival [49].

The common denominator obtained from these studies was that the price of ICIs was always the most prominent factor influencing the outcome. Lower price of ICIs could reduce the total cost of immunotherapy combined with chemotherapy, and therefore lower the ICER. The combination of immunotherapy and chemotherapy for ES-SCLC was cost-effective when the ICER was below the WTP threshold. Thus, lowering the price of ICIs (implementing patient assistance programs or paying health insurance) was the best option to improve the cost-effectiveness of the ICIs-combined treatment. We also noted that the proposed price reductions for ICIs in the included studies differed. The disparity in the results could be explained by various WTP thresholds or model designs across different countries, and the differences in administration, follow-up treatment costs, and discounts offered by pharmaceutical enterprises may also lead to different results. Furthermore, selecting patients who were sensitive to ICIs also provided a way to improve the cost-effectiveness of ICIs-combined treatment without adjusting for price [50, 51].

Our work has some limitations that should be addressed. Firstly, the number of the included studies is small. The fact that the clinical data in the included studies was collected retrospectively from seven published clinical trials instead of from patients in clinical practice raised questions about the generalizability of the results. Secondly, the utility values in the included studies were based on hypothesis or obtained from previously published literature, since they were not available from the published clinical trials, and may not be consistent with the actual real case. Thirdly, since the included studies were from different countries, and were analyzed from different perspectives using models, therefore, the costs, WTP thresholds, and model designs were different, which may have affected the results.

## Conclusion

ADEP and SEP were probably cost-effective treatments for ES-SCLC in China, and SEP could be cost-effective for patients with ES-SCLC in the U.S. when the WTP was $150,000.00. AEP was probably more cost-effective than DEP, NEP, IEP, and PEP in China when the WTP threshold was $31,313, but whether AEP was more cost-effective than EP alone remained unknown. Other treatments of immunotherapy combined with chemotherapy were not cost-effective for ES-SCLC. The most significant way to improve the cost-effectiveness of the combination of immunotherapy and chemotherapy for ES-SCLC was by reducing ICIs price (implementing patient assistance programs or paying medical insurance). Selecting patients who were sensitive to ICIs was also an alternative option to improve the cost-effectiveness of this combination treatment without adjusting for price.

## Supplementary Information

**Additional file 1.**

## Data Availability

The datasets used and/or analysed during the current study are available from the corresponding author on reasonable request.

## Abbreviations

ES-SCLC: Extensive-stage small-cell lung cancer
ICIs: Immune checkpoint inhibitors
EP: Platinum-etoposide
CHEERS: Consolidated Health Economic Evaluation Reporting Standards
LS-SCLC: Localized-stage small-cell lung cancer
PD-1: Programmed cell death protein 1
PD-L1: Programmed death-ligand 1
PRISMA: The Preferred Reporting Items for Systematic Reviews and Meta-Analyses Statement
CiCER: The Criteria for Cost(-Effectiveness) Review Outcomes
ISPOR: The International Society for Pharmacoeconomics and Outcomes Research
MeSH: Medical Subject Headings
ICER: Incremental cost-effectiveness ratio
WTP: Willingness-to-pay
ADEP: Adebrelimab plus platinum-etoposide
SEP: Serplulimab plus platinum-etoposide
NEP: Nivolumab plus platinum-etoposide
IEP: Ipilimumab plus platinum-etoposide
PEP: Pembrolizumab plus platinum-etoposide
AEP: Atezolizumab plus platinum-etoposide
DEP: Durvalumab plus platinum-etoposide
QALY: Quality-adjusted life year
PS: Partitioned survival
Lys: Life-years
OS: Overall survival
LYG: Life Year Gained
