# Supplementary material for: Cost-effectiveness of the combination of immunotherapy and chemotherapy for extensive-stage small-cell lung cancer: a systematic review
Source: BMC Health Serv Res. 2023 Jun 26;23:691. doi: 10.1186/s12913-023-09727-7 (PMC10294391; doi:10.1186/s12913-023-09727-7)
Supplement: Supplementary file 1 — Additional file 1. [file 12913_2023_9727_MOESM1_ESM.docx]

**Search strategy**

Search date: April 20, 2023

Databases searched: Web of Science, PubMed, EMBASE, and Cochrane Library

**Search Strategy in Web of science**

| Number | Query | Results |
| --- | --- | --- |
| #4 | #3 AND #2 AND #1 | 36 |
| #3 | Analys* Cost-Benefit (Topic) or Cost-Benefit Analys* (Topic) or Cost Benefit Analys* (Topic) or Analys*, Cost Benefit (Topic) or Cost Effectiveness (Topic) or Effectiveness, Cost (Topic) or Cost-Benefit Data (Topic) or Cost Benefit Data (Topic) or Data, Cost-Benefit (Topic) or Cost-Utility Analys* (Topic) or Analys*, Cost-Utility (Topic) or Cost Utility Analys* (Topic) or Economic Evaluatio* (Topic) or Evaluatio*, Economic (Topic) or Marginal Analys* (Topic) or Analys*, Marginal (Topic) or Cost Benefit (Topic) or Costs and Benefits (Topic) or Benefits and Costs (Topic) or Cost and Benefit (Topic) or Benefit and Cost (Topic) or Cost-Effectiveness Analysis (Topic) or Analysis, Cost-Effectiveness (Topic) or Cost Effectiveness Analysis (Topic) or cost consequence (Topic) or health care costs (Topic) | 1111361 |
| #2 | Chemotherapy (Topic) or Chemotherapies (Topic) | 1791696 |
| #1 | extensive-stage small-cell lung cancer (Topic) or extensive-stage small-cell lung cancers (Topic) | 1601 |

**36 Results**

**Search Strategy in PUBMED**

| Search number | Query | Search Details | Results |
| --- | --- | --- | --- |
| 6 | ((("Cost-Benefit Analysis"[Mesh]) OR (((((((((((((((((((((((((((Analys* Cost-Benefit) ) OR (Cost-Benefit Analys*)) OR (Cost Benefit Analys*)) OR (Analys*, Cost Benefit)) OR (Cost Effectiveness)) OR (Effectiveness, Cost)) OR (Cost-Benefit Data)) OR (Cost Benefit Data)) OR (Data, Cost-Benefit)) OR (Cost-Utility Analys*)) OR (Analys*, Cost-Utility)) OR (Cost Utility Analys*)) OR (Economic Evaluatio*)) OR (Evaluatio*, Economic)) OR (Marginal Analys*)) OR (Analys*, Marginal)) OR (Cost Benefit)) OR (Costs and Benefits)) OR (Benefits and Costs)) OR (Cost and Benefit)) OR (Benefit and Cost)) OR (Cost-Effectiveness Analysis)) OR (Analysis, Cost-Effectiveness)) OR (Cost Effectiveness Analysis)) OR (cost consequence)) OR (health care costs))) AND ((extensive-stage small-cell lung cancer) OR (extensive-stage small-cell lung cancers))) AND ((chemotherapy) OR (chemotherapies)) | ("Cost-Benefit Analysis"[MeSH Terms] OR (("analys*"[All Fields] AND ("Cost-Benefit Analysis"[MeSH Terms] OR ("cost benefit"[All Fields] AND "analysis"[All Fields]) OR "Cost-Benefit Analysis"[All Fields] OR ("cost"[All Fields] AND "benefit"[All Fields]) OR "cost benefit"[All Fields])) OR (("Cost-Benefit Analysis"[MeSH Terms] OR ("cost benefit"[All Fields] AND "analysis"[All Fields]) OR "Cost-Benefit Analysis"[All Fields] OR ("cost"[All Fields] AND "benefit"[All Fields]) OR "cost benefit"[All Fields]) AND "analys*"[All Fields]) OR (("Cost-Benefit Analysis"[MeSH Terms] OR ("cost benefit"[All Fields] AND "analysis"[All Fields]) OR "Cost-Benefit Analysis"[All Fields] OR ("cost"[All Fields] AND "benefit"[All Fields]) OR "cost benefit"[All Fields]) AND "analys*"[All Fields]) OR ("analys*"[All Fields] AND ("Cost-Benefit Analysis"[MeSH Terms] OR ("cost benefit"[All Fields] AND "analysis"[All Fields]) OR "Cost-Benefit Analysis"[All Fields] OR ("cost"[All Fields] AND "benefit"[All Fields]) OR "cost benefit"[All Fields])) OR ("Cost-Benefit Analysis"[MeSH Terms] OR ("cost benefit"[All Fields] AND "analysis"[All Fields]) OR "Cost-Benefit Analysis"[All Fields] OR ("cost"[All Fields] AND "effectiveness"[All Fields]) OR "cost effectiveness"[All Fields]) OR ("Cost-Benefit Analysis"[MeSH Terms] OR ("cost benefit"[All Fields] AND "analysis"[All Fields]) OR "Cost-Benefit Analysis"[All Fields] OR ("effectiveness"[All Fields] AND "cost"[All Fields]) OR "effectiveness cost"[All Fields]) OR ("Cost-Benefit Analysis"[MeSH Terms] OR ("cost benefit"[All Fields] AND "analysis"[All Fields]) OR "Cost-Benefit Analysis"[All Fields] OR ("cost"[All Fields] AND "benefit"[All Fields] AND "data"[All Fields]) OR "cost benefit data"[All Fields]) OR ("Cost-Benefit Analysis"[MeSH Terms] OR ("cost benefit"[All Fields] AND "analysis"[All Fields]) OR "Cost-Benefit Analysis"[All Fields] OR ("cost"[All Fields] AND "benefit"[All Fields] AND "data"[All Fields]) OR "cost benefit data"[All Fields]) OR ("Cost-Benefit Analysis"[MeSH Terms] OR ("cost benefit"[All Fields] AND "analysis"[All Fields]) OR "Cost-Benefit Analysis"[All Fields] OR ("data"[All Fields] AND "cost"[All Fields] AND "benefit"[All Fields]) OR "data cost benefit"[All Fields]) OR ("Cost-Utility"[All Fields] AND "analys*"[All Fields]) OR ("analys*"[All Fields] AND "Cost-Utility"[All Fields]) OR (("economics"[MeSH Subheading] OR "economics"[All Fields] OR "cost"[All Fields] OR "costs and cost analysis"[MeSH Terms] OR ("costs"[All Fields] AND "cost"[All Fields] AND "analysis"[All Fields]) OR "costs and cost analysis"[All Fields]) AND ("statistics and numerical data"[MeSH Subheading] OR ("statistics"[All Fields] AND "numerical"[All Fields] AND "data"[All Fields]) OR "statistics and numerical data"[All Fields] OR "utilization"[All Fields] OR "utilisation"[All Fields] OR "utilisations"[All Fields] OR "utilise"[All Fields] OR "utilised"[All Fields] OR "utilises"[All Fields] OR "utilising"[All Fields] OR "utilities"[All Fields] OR "utility"[All Fields] OR "utilizations"[All Fields] OR "utilize"[All Fields] OR "utilized"[All Fields] OR "utilizer"[All Fields] OR "utilizers"[All Fields] OR "utilizes"[All Fields] OR "utilizing"[All Fields]) AND "analys*"[All Fields]) OR (("economical"[All Fields] OR "economics"[MeSH Terms] OR "economics"[All Fields] OR "economic"[All Fields] OR "economically"[All Fields] OR "economics"[MeSH Subheading] OR "economization"[All Fields] OR "economize"[All Fields] OR "economized"[All Fields] OR "economizes"[All Fields] OR "economizing"[All Fields]) AND "evaluatio*"[All Fields]) OR ("evaluatio*"[All Fields] AND ("economical"[All Fields] OR "economics"[MeSH Terms] OR "economics"[All Fields] OR "economic"[All Fields] OR "economically"[All Fields] OR "economics"[MeSH Subheading] OR "economization"[All Fields] OR "economize"[All Fields] OR "economized"[All Fields] OR "economizes"[All Fields] OR "economizing"[All Fields])) OR (("margin s"[All Fields] OR "marginal"[All Fields] OR "marginals"[All Fields] OR "margined"[All Fields] OR "margins of excision"[MeSH Terms] OR ("margins"[All Fields] AND "excision"[All Fields]) OR "margins of excision"[All Fields] OR "margin"[All Fields] OR "margins"[All Fields]) AND "analys*"[All Fields]) OR ("analys*"[All Fields] AND ("margin s"[All Fields] OR "marginal"[All Fields] OR "marginals"[All Fields] OR "margined"[All Fields] OR "margins of excision"[MeSH Terms] OR ("margins"[All Fields] AND "excision"[All Fields]) OR "margins of excision"[All Fields] OR "margin"[All Fields] OR "margins"[All Fields])) OR ("Cost-Benefit Analysis"[MeSH Terms] OR ("cost benefit"[All Fields] AND "analysis"[All Fields]) OR "Cost-Benefit Analysis"[All Fields] OR ("cost"[All Fields] AND "benefit"[All Fields]) OR "cost benefit"[All Fields]) OR ("Cost-Benefit Analysis"[MeSH Terms] OR ("cost benefit"[All Fields] AND "analysis"[All Fields]) OR "Cost-Benefit Analysis"[All Fields] OR ("costs"[All Fields] AND "benefits"[All Fields]) OR "costs and benefits"[All Fields]) OR ("Cost-Benefit Analysis"[MeSH Terms] OR ("cost benefit"[All Fields] AND "analysis"[All Fields]) OR "Cost-Benefit Analysis"[All Fields] OR ("benefits"[All Fields] AND "costs"[All Fields]) OR "benefits and costs"[All Fields]) OR ("Cost-Benefit Analysis"[MeSH Terms] OR ("cost benefit"[All Fields] AND "analysis"[All Fields]) OR "Cost-Benefit Analysis"[All Fields] OR ("cost"[All Fields] AND "benefit"[All Fields]) OR "cost and benefit"[All Fields]) OR ("Cost-Benefit Analysis"[MeSH Terms] OR ("cost benefit"[All Fields] AND "analysis"[All Fields]) OR "Cost-Benefit Analysis"[All Fields] OR ("benefit"[All Fields] AND "cost"[All Fields]) OR "benefit and cost"[All Fields]) OR ("Cost-Benefit Analysis"[MeSH Terms] OR ("cost benefit"[All Fields] AND "analysis"[All Fields]) OR "Cost-Benefit Analysis"[All Fields] OR ("cost"[All Fields] AND "effectiveness"[All Fields] AND "analysis"[All Fields]) OR "cost effectiveness analysis"[All Fields]) OR ("Cost-Benefit Analysis"[MeSH Terms] OR ("cost benefit"[All Fields] AND "analysis"[All Fields]) OR "Cost-Benefit Analysis"[All Fields] OR ("analysis"[All Fields] AND "cost"[All Fields] AND "effectiveness"[All Fields]) OR "analysis cost effectiveness"[All Fields]) OR ("Cost-Benefit Analysis"[MeSH Terms] OR ("cost benefit"[All Fields] AND "analysis"[All Fields]) OR "Cost-Benefit Analysis"[All Fields] OR ("cost"[All Fields] AND "effectiveness"[All Fields] AND "analysis"[All Fields]) OR "cost effectiveness analysis"[All Fields]) OR (("economics"[MeSH Subheading] OR "economics"[All Fields] OR "cost"[All Fields] OR "costs and cost analysis"[MeSH Terms] OR ("costs"[All Fields] AND "cost"[All Fields] AND "analysis"[All Fields]) OR "costs and cost analysis"[All Fields]) AND ("consequence"[All Fields] OR "consequences"[All Fields] OR "consequent"[All Fields] OR "consequently"[All Fields] OR "consequents"[All Fields])) OR ("health care costs"[MeSH Terms] OR ("health"[All Fields] AND "care"[All Fields] AND "costs"[All Fields]) OR "health care costs"[All Fields]))) AND (("extensive-stage"[All Fields] AND ("small cell lung carcinoma"[MeSH Terms] OR ("small"[All Fields] AND "cell"[All Fields] AND "lung"[All Fields] AND "carcinoma"[All Fields]) OR "small cell lung carcinoma"[All Fields] OR ("small"[All Fields] AND "cell"[All Fields] AND "lung"[All Fields] AND "cancer"[All Fields]) OR "small cell lung cancer"[All Fields])) OR ("extensive-stage"[All Fields] AND "small-cell"[All Fields] AND ("lung neoplasms"[MeSH Terms] OR ("lung"[All Fields] AND "neoplasms"[All Fields]) OR "lung neoplasms"[All Fields] OR ("lung"[All Fields] AND "cancers"[All Fields]) OR "lung cancers"[All Fields]))) AND ("chemotherapy s"[All Fields] OR "drug therapy"[MeSH Terms] OR ("drug"[All Fields] AND "therapy"[All Fields]) OR "drug therapy"[All Fields] OR "chemotherapies"[All Fields] OR "drug therapy"[MeSH Subheading] OR "chemotherapy"[All Fields] OR ("chemotherapy s"[All Fields] OR "drug therapy"[MeSH Terms] OR ("drug"[All Fields] AND "therapy"[All Fields]) OR "drug therapy"[All Fields] OR "chemotherapies"[All Fields] OR "drug therapy"[MeSH Subheading] OR "chemotherapy"[All Fields])) | 28 |
| 5 | (chemotherapy) OR (chemotherapies) | "chemotherapy s"[All Fields] OR "drug therapy"[MeSH Terms] OR ("drug"[All Fields] AND "therapy"[All Fields]) OR "drug therapy"[All Fields] OR "chemotherapies"[All Fields] OR "drug therapy"[MeSH Subheading] OR "chemotherapy"[All Fields] OR ("chemotherapy s"[All Fields] OR "drug therapy"[MeSH Terms] OR ("drug"[All Fields] AND "therapy"[All Fields]) OR "drug therapy"[All Fields] OR "chemotherapies"[All Fields] OR "drug therapy"[MeSH Subheading] OR "chemotherapy"[All Fields]) | 3669139 |
| 4 | (extensive-stage small-cell lung cancer) OR (extensive-stage small-cell lung cancers) | ("extensive-stage"[All Fields] AND ("small cell lung carcinoma"[MeSH Terms] OR ("small"[All Fields] AND "cell"[All Fields] AND "lung"[All Fields] AND "carcinoma"[All Fields]) OR "small cell lung carcinoma"[All Fields] OR ("small"[All Fields] AND "cell"[All Fields] AND "lung"[All Fields] AND "cancer"[All Fields]) OR "small cell lung cancer"[All Fields])) OR ("extensive-stage"[All Fields] AND "small-cell"[All Fields] AND ("lung neoplasms"[MeSH Terms] OR ("lung"[All Fields] AND "neoplasms"[All Fields]) OR "lung neoplasms"[All Fields] OR ("lung"[All Fields] AND "cancers"[All Fields]) OR "lung cancers"[All Fields])) | 1,066 |
| 3 | ("Cost-Benefit Analysis"[Mesh]) OR (((((((((((((((((((((((((((Analys* Cost-Benefit) ) OR (Cost-Benefit Analys*)) OR (Cost Benefit Analys*)) OR (Analys*, Cost Benefit)) OR (Cost Effectiveness)) OR (Effectiveness, Cost)) OR (Cost-Benefit Data)) OR (Cost Benefit Data)) OR (Data, Cost-Benefit)) OR (Cost-Utility Analys*)) OR (Analys*, Cost-Utility)) OR (Cost Utility Analys*)) OR (Economic Evaluatio*)) OR (Evaluatio*, Economic)) OR (Marginal Analys*)) OR (Analys*, Marginal)) OR (Cost Benefit)) OR (Costs and Benefits)) OR (Benefits and Costs)) OR (Cost and Benefit)) OR (Benefit and Cost)) OR (Cost-Effectiveness Analysis)) OR (Analysis, Cost-Effectiveness)) OR (Cost Effectiveness Analysis)) OR (cost consequence)) OR (health care costs)) | "Cost-Benefit Analysis"[MeSH Terms] OR (("analys*"[All Fields] AND ("Cost-Benefit Analysis"[MeSH Terms] OR ("cost benefit"[All Fields] AND "analysis"[All Fields]) OR "Cost-Benefit Analysis"[All Fields] OR ("cost"[All Fields] AND "benefit"[All Fields]) OR "cost benefit"[All Fields])) OR (("Cost-Benefit Analysis"[MeSH Terms] OR ("cost benefit"[All Fields] AND "analysis"[All Fields]) OR "Cost-Benefit Analysis"[All Fields] OR ("cost"[All Fields] AND "benefit"[All Fields]) OR "cost benefit"[All Fields]) AND "analys*"[All Fields]) OR (("Cost-Benefit Analysis"[MeSH Terms] OR ("cost benefit"[All Fields] AND "analysis"[All Fields]) OR "Cost-Benefit Analysis"[All Fields] OR ("cost"[All Fields] AND "benefit"[All Fields]) OR "cost benefit"[All Fields]) AND "analys*"[All Fields]) OR ("analys*"[All Fields] AND ("Cost-Benefit Analysis"[MeSH Terms] OR ("cost benefit"[All Fields] AND "analysis"[All Fields]) OR "Cost-Benefit Analysis"[All Fields] OR ("cost"[All Fields] AND "benefit"[All Fields]) OR "cost benefit"[All Fields])) OR ("Cost-Benefit Analysis"[MeSH Terms] OR ("cost benefit"[All Fields] AND "analysis"[All Fields]) OR "Cost-Benefit Analysis"[All Fields] OR ("cost"[All Fields] AND "effectiveness"[All Fields]) OR "cost effectiveness"[All Fields]) OR ("Cost-Benefit Analysis"[MeSH Terms] OR ("cost benefit"[All Fields] AND "analysis"[All Fields]) OR "Cost-Benefit Analysis"[All Fields] OR ("effectiveness"[All Fields] AND "cost"[All Fields]) OR "effectiveness cost"[All Fields]) OR ("Cost-Benefit Analysis"[MeSH Terms] OR ("cost benefit"[All Fields] AND "analysis"[All Fields]) OR "Cost-Benefit Analysis"[All Fields] OR ("cost"[All Fields] AND "benefit"[All Fields] AND "data"[All Fields]) OR "cost benefit data"[All Fields]) OR ("Cost-Benefit Analysis"[MeSH Terms] OR ("cost benefit"[All Fields] AND "analysis"[All Fields]) OR "Cost-Benefit Analysis"[All Fields] OR ("cost"[All Fields] AND "benefit"[All Fields] AND "data"[All Fields]) OR "cost benefit data"[All Fields]) OR ("Cost-Benefit Analysis"[MeSH Terms] OR ("cost benefit"[All Fields] AND "analysis"[All Fields]) OR "Cost-Benefit Analysis"[All Fields] OR ("data"[All Fields] AND "cost"[All Fields] AND "benefit"[All Fields]) OR "data cost benefit"[All Fields]) OR ("Cost-Utility"[All Fields] AND "analys*"[All Fields]) OR ("analys*"[All Fields] AND "Cost-Utility"[All Fields]) OR (("economics"[MeSH Subheading] OR "economics"[All Fields] OR "cost"[All Fields] OR "costs and cost analysis"[MeSH Terms] OR ("costs"[All Fields] AND "cost"[All Fields] AND "analysis"[All Fields]) OR "costs and cost analysis"[All Fields]) AND ("statistics and numerical data"[MeSH Subheading] OR ("statistics"[All Fields] AND "numerical"[All Fields] AND "data"[All Fields]) OR "statistics and numerical data"[All Fields] OR "utilization"[All Fields] OR "utilisation"[All Fields] OR "utilisations"[All Fields] OR "utilise"[All Fields] OR "utilised"[All Fields] OR "utilises"[All Fields] OR "utilising"[All Fields] OR "utilities"[All Fields] OR "utility"[All Fields] OR "utilizations"[All Fields] OR "utilize"[All Fields] OR "utilized"[All Fields] OR "utilizer"[All Fields] OR "utilizers"[All Fields] OR "utilizes"[All Fields] OR "utilizing"[All Fields]) AND "analys*"[All Fields]) OR (("economical"[All Fields] OR "economics"[MeSH Terms] OR "economics"[All Fields] OR "economic"[All Fields] OR "economically"[All Fields] OR "economics"[MeSH Subheading] OR "economization"[All Fields] OR "economize"[All Fields] OR "economized"[All Fields] OR "economizes"[All Fields] OR "economizing"[All Fields]) AND "evaluatio*"[All Fields]) OR ("evaluatio*"[All Fields] AND ("economical"[All Fields] OR "economics"[MeSH Terms] OR "economics"[All Fields] OR "economic"[All Fields] OR "economically"[All Fields] OR "economics"[MeSH Subheading] OR "economization"[All Fields] OR "economize"[All Fields] OR "economized"[All Fields] OR "economizes"[All Fields] OR "economizing"[All Fields])) OR (("margin s"[All Fields] OR "marginal"[All Fields] OR "marginals"[All Fields] OR "margined"[All Fields] OR "margins of excision"[MeSH Terms] OR ("margins"[All Fields] AND "excision"[All Fields]) OR "margins of excision"[All Fields] OR "margin"[All Fields] OR "margins"[All Fields]) AND "analys*"[All Fields]) OR ("analys*"[All Fields] AND ("margin s"[All Fields] OR "marginal"[All Fields] OR "marginals"[All Fields] OR "margined"[All Fields] OR "margins of excision"[MeSH Terms] OR ("margins"[All Fields] AND "excision"[All Fields]) OR "margins of excision"[All Fields] OR "margin"[All Fields] OR "margins"[All Fields])) OR ("Cost-Benefit Analysis"[MeSH Terms] OR ("cost benefit"[All Fields] AND "analysis"[All Fields]) OR "Cost-Benefit Analysis"[All Fields] OR ("cost"[All Fields] AND "benefit"[All Fields]) OR "cost benefit"[All Fields]) OR ("Cost-Benefit Analysis"[MeSH Terms] OR ("cost benefit"[All Fields] AND "analysis"[All Fields]) OR "Cost-Benefit Analysis"[All Fields] OR ("costs"[All Fields] AND "benefits"[All Fields]) OR "costs and benefits"[All Fields]) OR ("Cost-Benefit Analysis"[MeSH Terms] OR ("cost benefit"[All Fields] AND "analysis"[All Fields]) OR "Cost-Benefit Analysis"[All Fields] OR ("benefits"[All Fields] AND "costs"[All Fields]) OR "benefits and costs"[All Fields]) OR ("Cost-Benefit Analysis"[MeSH Terms] OR ("cost benefit"[All Fields] AND "analysis"[All Fields]) OR "Cost-Benefit Analysis"[All Fields] OR ("cost"[All Fields] AND "benefit"[All Fields]) OR "cost and benefit"[All Fields]) OR ("Cost-Benefit Analysis"[MeSH Terms] OR ("cost benefit"[All Fields] AND "analysis"[All Fields]) OR "Cost-Benefit Analysis"[All Fields] OR ("benefit"[All Fields] AND "cost"[All Fields]) OR "benefit and cost"[All Fields]) OR ("Cost-Benefit Analysis"[MeSH Terms] OR ("cost benefit"[All Fields] AND "analysis"[All Fields]) OR "Cost-Benefit Analysis"[All Fields] OR ("cost"[All Fields] AND "effectiveness"[All Fields] AND "analysis"[All Fields]) OR "cost effectiveness analysis"[All Fields]) OR ("Cost-Benefit Analysis"[MeSH Terms] OR ("cost benefit"[All Fields] AND "analysis"[All Fields]) OR "Cost-Benefit Analysis"[All Fields] OR ("analysis"[All Fields] AND "cost"[All Fields] AND "effectiveness"[All Fields]) OR "analysis cost effectiveness"[All Fields]) OR ("Cost-Benefit Analysis"[MeSH Terms] OR ("cost benefit"[All Fields] AND "analysis"[All Fields]) OR "Cost-Benefit Analysis"[All Fields] OR ("cost"[All Fields] AND "effectiveness"[All Fields] AND "analysis"[All Fields]) OR "cost effectiveness analysis"[All Fields]) OR (("economics"[MeSH Subheading] OR "economics"[All Fields] OR "cost"[All Fields] OR "costs and cost analysis"[MeSH Terms] OR ("costs"[All Fields] AND "cost"[All Fields] AND "analysis"[All Fields]) OR "costs and cost analysis"[All Fields]) AND ("consequence"[All Fields] OR "consequences"[All Fields] OR "consequent"[All Fields] OR "consequently"[All Fields] OR "consequents"[All Fields])) OR ("health care costs"[MeSH Terms] OR ("health"[All Fields] AND "care"[All Fields] AND "costs"[All Fields]) OR "health care costs"[All Fields])) | 488,904 |
| 2 | ((((((((((((((((((((((((((Analys* Cost-Benefit) ) OR (Cost-Benefit Analys*)) OR (Cost Benefit Analys*)) OR (Analys*, Cost Benefit)) OR (Cost Effectiveness)) OR (Effectiveness, Cost)) OR (Cost-Benefit Data)) OR (Cost Benefit Data)) OR (Data, Cost-Benefit)) OR (Cost-Utility Analys*)) OR (Analys*, Cost-Utility)) OR (Cost Utility Analys*)) OR (Economic Evaluatio*)) OR (Evaluatio*, Economic)) OR (Marginal Analys*)) OR (Analys*, Marginal)) OR (Cost Benefit)) OR (Costs and Benefits)) OR (Benefits and Costs)) OR (Cost and Benefit)) OR (Benefit and Cost)) OR (Cost-Effectiveness Analysis)) OR (Analysis, Cost-Effectiveness)) OR (Cost Effectiveness Analysis)) OR (cost consequence)) OR (health care costs) | ("analys*"[All Fields] AND ("cost benefit analysis"[MeSH Terms] OR ("cost benefit"[All Fields] AND "analysis"[All Fields]) OR "cost benefit analysis"[All Fields] OR ("cost"[All Fields] AND "benefit"[All Fields]) OR "cost benefit"[All Fields])) OR (("cost benefit analysis"[MeSH Terms] OR ("cost benefit"[All Fields] AND "analysis"[All Fields]) OR "cost benefit analysis"[All Fields] OR ("cost"[All Fields] AND "benefit"[All Fields]) OR "cost benefit"[All Fields]) AND "analys*"[All Fields]) OR (("cost benefit analysis"[MeSH Terms] OR ("cost benefit"[All Fields] AND "analysis"[All Fields]) OR "cost benefit analysis"[All Fields] OR ("cost"[All Fields] AND "benefit"[All Fields]) OR "cost benefit"[All Fields]) AND "analys*"[All Fields]) OR ("analys*"[All Fields] AND ("cost benefit analysis"[MeSH Terms] OR ("cost benefit"[All Fields] AND "analysis"[All Fields]) OR "cost benefit analysis"[All Fields] OR ("cost"[All Fields] AND "benefit"[All Fields]) OR "cost benefit"[All Fields])) OR ("cost benefit analysis"[MeSH Terms] OR ("cost benefit"[All Fields] AND "analysis"[All Fields]) OR "cost benefit analysis"[All Fields] OR ("cost"[All Fields] AND "effectiveness"[All Fields]) OR "cost effectiveness"[All Fields]) OR ("cost benefit analysis"[MeSH Terms] OR ("cost benefit"[All Fields] AND "analysis"[All Fields]) OR "cost benefit analysis"[All Fields] OR ("effectiveness"[All Fields] AND "cost"[All Fields]) OR "effectiveness cost"[All Fields]) OR ("cost benefit analysis"[MeSH Terms] OR ("cost benefit"[All Fields] AND "analysis"[All Fields]) OR "cost benefit analysis"[All Fields] OR ("cost"[All Fields] AND "benefit"[All Fields] AND "data"[All Fields]) OR "cost benefit data"[All Fields]) OR ("cost benefit analysis"[MeSH Terms] OR ("cost benefit"[All Fields] AND "analysis"[All Fields]) OR "cost benefit analysis"[All Fields] OR ("cost"[All Fields] AND "benefit"[All Fields] AND "data"[All Fields]) OR "cost benefit data"[All Fields]) OR ("cost benefit analysis"[MeSH Terms] OR ("cost benefit"[All Fields] AND "analysis"[All Fields]) OR "cost benefit analysis"[All Fields] OR ("data"[All Fields] AND "cost"[All Fields] AND "benefit"[All Fields]) OR "data cost benefit"[All Fields]) OR ("Cost-Utility"[All Fields] AND "analys*"[All Fields]) OR ("analys*"[All Fields] AND "Cost-Utility"[All Fields]) OR (("economics"[MeSH Subheading] OR "economics"[All Fields] OR "cost"[All Fields] OR "costs and cost analysis"[MeSH Terms] OR ("costs"[All Fields] AND "cost"[All Fields] AND "analysis"[All Fields]) OR "costs and cost analysis"[All Fields]) AND ("statistics and numerical data"[MeSH Subheading] OR ("statistics"[All Fields] AND "numerical"[All Fields] AND "data"[All Fields]) OR "statistics and numerical data"[All Fields] OR "utilization"[All Fields] OR "utilisation"[All Fields] OR "utilisations"[All Fields] OR "utilise"[All Fields] OR "utilised"[All Fields] OR "utilises"[All Fields] OR "utilising"[All Fields] OR "utilities"[All Fields] OR "utility"[All Fields] OR "utilizations"[All Fields] OR "utilize"[All Fields] OR "utilized"[All Fields] OR "utilizer"[All Fields] OR "utilizers"[All Fields] OR "utilizes"[All Fields] OR "utilizing"[All Fields]) AND "analys*"[All Fields]) OR (("economical"[All Fields] OR "economics"[MeSH Terms] OR "economics"[All Fields] OR "economic"[All Fields] OR "economically"[All Fields] OR "economics"[MeSH Subheading] OR "economization"[All Fields] OR "economize"[All Fields] OR "economized"[All Fields] OR "economizes"[All Fields] OR "economizing"[All Fields]) AND "evaluatio*"[All Fields]) OR ("evaluatio*"[All Fields] AND ("economical"[All Fields] OR "economics"[MeSH Terms] OR "economics"[All Fields] OR "economic"[All Fields] OR "economically"[All Fields] OR "economics"[MeSH Subheading] OR "economization"[All Fields] OR "economize"[All Fields] OR "economized"[All Fields] OR "economizes"[All Fields] OR "economizing"[All Fields])) OR (("margin s"[All Fields] OR "marginal"[All Fields] OR "marginals"[All Fields] OR "margined"[All Fields] OR "margins of excision"[MeSH Terms] OR ("margins"[All Fields] AND "excision"[All Fields]) OR "margins of excision"[All Fields] OR "margin"[All Fields] OR "margins"[All Fields]) AND "analys*"[All Fields]) OR ("analys*"[All Fields] AND ("margin s"[All Fields] OR "marginal"[All Fields] OR "marginals"[All Fields] OR "margined"[All Fields] OR "margins of excision"[MeSH Terms] OR ("margins"[All Fields] AND "excision"[All Fields]) OR "margins of excision"[All Fields] OR "margin"[All Fields] OR "margins"[All Fields])) OR ("cost benefit analysis"[MeSH Terms] OR ("cost benefit"[All Fields] AND "analysis"[All Fields]) OR "cost benefit analysis"[All Fields] OR ("cost"[All Fields] AND "benefit"[All Fields]) OR "cost benefit"[All Fields]) OR ("cost benefit analysis"[MeSH Terms] OR ("cost benefit"[All Fields] AND "analysis"[All Fields]) OR "cost benefit analysis"[All Fields] OR ("costs"[All Fields] AND "benefits"[All Fields]) OR "costs and benefits"[All Fields]) OR ("cost benefit analysis"[MeSH Terms] OR ("cost benefit"[All Fields] AND "analysis"[All Fields]) OR "cost benefit analysis"[All Fields] OR ("benefits"[All Fields] AND "costs"[All Fields]) OR "benefits and costs"[All Fields]) OR ("cost benefit analysis"[MeSH Terms] OR ("cost benefit"[All Fields] AND "analysis"[All Fields]) OR "cost benefit analysis"[All Fields] OR ("cost"[All Fields] AND "benefit"[All Fields]) OR "cost and benefit"[All Fields]) OR ("cost benefit analysis"[MeSH Terms] OR ("cost benefit"[All Fields] AND "analysis"[All Fields]) OR "cost benefit analysis"[All Fields] OR ("benefit"[All Fields] AND "cost"[All Fields]) OR "benefit and cost"[All Fields]) OR ("cost benefit analysis"[MeSH Terms] OR ("cost benefit"[All Fields] AND "analysis"[All Fields]) OR "cost benefit analysis"[All Fields] OR ("cost"[All Fields] AND "effectiveness"[All Fields] AND "analysis"[All Fields]) OR "cost effectiveness analysis"[All Fields]) OR ("cost benefit analysis"[MeSH Terms] OR ("cost benefit"[All Fields] AND "analysis"[All Fields]) OR "cost benefit analysis"[All Fields] OR ("analysis"[All Fields] AND "cost"[All Fields] AND "effectiveness"[All Fields]) OR "analysis cost effectiveness"[All Fields]) OR ("cost benefit analysis"[MeSH Terms] OR ("cost benefit"[All Fields] AND "analysis"[All Fields]) OR "cost benefit analysis"[All Fields] OR ("cost"[All Fields] AND "effectiveness"[All Fields] AND "analysis"[All Fields]) OR "cost effectiveness analysis"[All Fields]) OR (("economics"[MeSH Subheading] OR "economics"[All Fields] OR "cost"[All Fields] OR "costs and cost analysis"[MeSH Terms] OR ("costs"[All Fields] AND "cost"[All Fields] AND "analysis"[All Fields]) OR "costs and cost analysis"[All Fields]) AND ("consequence"[All Fields] OR "consequences"[All Fields] OR "consequent"[All Fields] OR "consequently"[All Fields] OR "consequents"[All Fields])) OR ("health care costs"[MeSH Terms] OR ("health"[All Fields] AND "care"[All Fields] AND "costs"[All Fields]) OR "health care costs"[All Fields]) | 488,904 |
| 1 | "Cost-Benefit Analysis"[Mesh] | "Cost-Benefit Analysis"[MeSH Terms] | 88,107 |

**28 Results**

**Search Strategy in Embase**

| No. | Query | Results |
| --- | --- | --- |
| #8 | #3 AND #6 AND #7 | 36 |
| #7 | 'extensive stage' AND 'small cell' AND lung AND cancer OR ('extensive stage' AND 'small cell' AND lung AND cancers) | 1929 |
| #6 | #4 OR #5 | 1104897 |
| #5 | chemotherapy OR chemotherapies | 1015329 |
| #4 | 'chemotherapy'/exp | 743223 |
| #3 | #1 OR #2 | 543705 |
| #2 | analys* AND 'cost benefit' OR ('cost benefit' AND analys*) OR (cost AND benefit AND analys*) OR (analys*, AND cost AND benefit) OR (cost AND effectiveness) OR (effectiveness, AND cost) OR ('cost benefit' AND data) OR (cost AND benefit AND data) OR (data, AND 'cost benefit') OR ('cost utility' AND analys*) OR (analys*, AND 'cost utility') OR (cost AND utility AND analys*) OR (economic AND evaluatio*) OR (evaluatio*, AND economic) OR (marginal AND analys*) OR (analys*, AND marginal) OR (costs AND benefits) OR (benefits AND costs) OR (cost AND benefit) OR (benefit AND cost) OR ('cost effectiveness' AND analysis) OR (analysis, AND 'cost effectiveness') OR (cost AND effectiveness AND analysis) OR (cost AND consequence) OR (health AND care AND costs) | 543705 |
| #1 | 'cost benefit analysis'/exp | 89083 |

**36 Results**

**Search Strategy in the Cochrane Library**

| Number | Query | Results |
| --- | --- | --- |
| #11 | #1 and #2 and #10 | 5 |
| #10 | #3 or #4 or #5 or #6 or #7 or #8 or #9 | 49395 |
| #9 | (health care costs):ti,ab,kw | 15063 |
| #8 | (Benefit and Cost):ti,ab,kw OR (Cost-Effectiveness Analysis):ti,ab,kw OR (Analysis, Cost-Effectiveness):ti,ab,kw OR (Cost Effectiveness Analysis):ti,ab,kw OR (cost consequence):ti,ab,kw | 28211 |
| #7 | (Analys*, Marginal):ti,ab,kw OR (Cost Benefit):ti,ab,kw OR (Costs and Benefits):ti,ab,kw OR (Benefits and Costs):ti,ab,kw OR (Cost and Benefit):ti,ab,kw | 18042 |
| #6 | (Analys*, Cost-Utility):ti,ab,kw OR (Cost Utility Analys*):ti,ab,kw OR (Economic Evaluatio*):ti,ab,kw OR (Evaluatio*, Economic):ti,ab,kw OR (Marginal Analys*):ti,ab,kw | 11987 |
| #5 | (Effectiveness, Cost):ti,ab,kw OR (Cost-Benefit Data):ti,ab,kw OR (Cost Benefit Data):ti,ab,kw OR (Data, Cost-Benefit):ti,ab,kw OR (Cost-Utility Analys*):ti,ab,kw | 32421 |
| #4 | (Analys* Cost-Benefit):ti,ab,kw OR (Cost-Benefit Analys*):ti,ab,kw OR (Cost Benefit Analys*):ti,ab,kw OR (Analys*, Cost Benefit):ti,ab,kw OR (Cost Effectiveness):ti,ab,kw | 34554 |
| #3 | MeSH descriptor: [Cost-Benefit Analysis] explode all trees | 7536 |
| #2 | (extensive-stage small-cell lung cancer):ti,ab,kw OR (extensive-stage small-cell lung cancers):ti,ab,kw | 511 |
| #1 | (Chemotherapy):ti,ab,kw OR (Chemotherapies):ti,ab,kw | 81731 |

**5 Results**
